# Influence of Spontaneous and Mechanical Ventilation on Frequency-Based Measures of Heart Rate Variability

**DOI:** 10.1155/2021/8709262

**Published:** 2021-12-26

**Authors:** Khlood Bubshait, Yasmine Alabbasi

**Affiliations:** ^1^Fundamental of Nursing Department, Imam Abdulrahman Bin Faisal University, P.O. Box 1982, Dammam, Eastern Province 32441, Saudi Arabia; ^2^Maternity and Child Health Nursing Department, College of Nursing, Princess Nourah Bint Abdulrahman University, Riyadh, Saudi Arabia

## Abstract

Frequency-based measures of heart rate variability have been shown to be a useful physiological marker in both clinical and research settings providing insight into the functioning of the autonomic nervous system. Ongoing interactions between the autonomic nervous system control of the heart and lung occurs during each ventilation cycle because of their anatomical position within the closed thoracic cavity. Mechanical ventilation and subsequent removal change the normal ventilator mechanics producing alterations in the tidal volume, intrathoracic pressure, and oxygen delivery. A noninvasive method called heart rate variability (HRV) can be used to evaluate this interaction during ventilation and can be quantified by applying frequency-based measures of the variability between heartbeats. Although HRV is a reliable method to measure alteration of the autonomic nervous system (ANS) function and cardiopulmonary interaction, there have been limited reports concerning the changes in the frequency-based measure of HRV during both spontaneous and mechanical ventilation. The purpose of this methodological study is therefore to describe the physiological influence of both spontaneous and mechanical ventilation on frequency-based measures of HRV.

## 1. Introduction

Heart rate variability (HRV) is a noninvasive method to evaluate the alteration of autonomic nervous system (ANS) function and cardiopulmonary interaction [1]. HRV analyzes fluctuations of time intervals between heartbeats due to the joint action of the sympathetic and parasympathetic branches of the ANS [1, 2].

HRV analysis is used as a measure of cardiac ANS regulation [3]. HRV can be measured by using linear methods such as time-based or frequency-based measures (power spectral analysis) [1–3]. The frequency-based measure of HRV includes major components like the total spectrum or frequency (TF), low-frequency (LF) power (reflects LF power may instead be related to baroreflex modulation of cardiac autonomic outflows), high-frequency (HF) power (reflects the parasympathetic nervous system), and ratio between LF/HF power (depends upon the assumption that physiological interventions always elicit reciprocal changes in parasympathetic and sympathetic nerve activity and other related factors such as chemoreceptor activation) [4, 5]. The frequency-based measure, particularly the HF power, is more sensitive to respiration and is known to change during different respiratory patterns [6]. The effect of respiration on HRV is called respiratory sinus arrhythmia (RSA) [7]. Therefore, the respiratory pattern is one of the confounders that may affect the measures of HRV including HF, LF, and HF/LF ratio (particularly frequency-based measure).

The benefit of frequency-based method or spectral analysis of HRV is that it can identify different arrhythmias during different types of ventilation (spontaneous, MV, and weaning process) on adults, children, and high-risk infants [8]. Moreover, the ratio of LF to HF appears to be a sensitive measure of the ANS response to a sudden change in cardiovascular control, and this ratio represents an evaluation of the ANS balance [9].

RSA is a marker for ANS control (vagal tone) and contributes to improved gas exchange during mechanical ventilation (MV) [7, 10, 11]. Respiratory parameters, including tidal volume (VT) and intrathoracic pressure (ITP), are the most common confounders seen among patients in intensive care units (ICUs) [10]. For instance, patients with different modes of MV experience alteration in hemodynamic status and cardiovascular stability because of changes in ITP and VT [12]. Consequently, this alteration might affect the ANS, which may be reflected by the frequency-based measure of HRV (RSA) [12].

The influence of MV on HRV has been studied in nonhuman subjects such as canine models and human subjects, including newborn babies, children with brain death, and healthy young adults placed on sedation and paralysis [13–16]. The investigators found in both populations of nonhuman and human subjects that there was a significant increase in LF power and decrease in HF power or RSA with response to a combination of pressure support and continuous positive airway pressure (CPAP) [13–16].

It is important to know that breathing is a form of exercise, and normal respiratory frequency is the main factor maintaining the balance of the inspiration/expiration ratio (I : E ratio) [17]. Furthermore, studies on the effects of respiratory phase time ratio have reported a tendency for baroreflex (baroreceptor reflex is one of the body's homeostatic mechanisms that helps to maintain blood pressure at nearly constant levels) sensitivity and HRV amplitude to increase when the inspiration/expiration ratio is 1/1 during slow breathing at 0.1 Hz [17]. Patients on different modes of MV, particularly positive-mode ventilation, commonly experience I : E ratio of 1 : 1 due to decreased functional residual capacity, increased muscle resistance, and collapse is common [18]. As a result, longer than normal inspiratory times may be required for the alveoli to reach inspiratory equilibrium [17]. Expiratory pauses with no gas flow will not contribute to ventilation and may contribute to collapse. All the works of respiration are done by the MV machine [17, 18].

Despite the recognition that different types of MV are associated with alteration in the ANS, there have been limited reports concerning the changes in the frequency-based measure of HRV, which is a reliable method to detect ANS activity, particularly during MV [19]. Therefore, this review attempts to fulfill an important gap in the evidence base for the physiological changes in frequency-based measure of HRV among patients receiving different types of MV.

The purpose of this research study is to describe the physiological influence of spontaneous and MV on frequency-based measures of HRV. In this study, the difference between spontaneous respiration and MV was described. Additionally, a deep physiological review of autonomic tone, cardiovascular effects during spontaneous respiration, and HRV changes during spontaneous respiration, MV, and weaning from MV was described and illustrated by using figures.

### 1.1. Spontaneous Respiration versus Mechanical Ventilation

**Figure 1:**
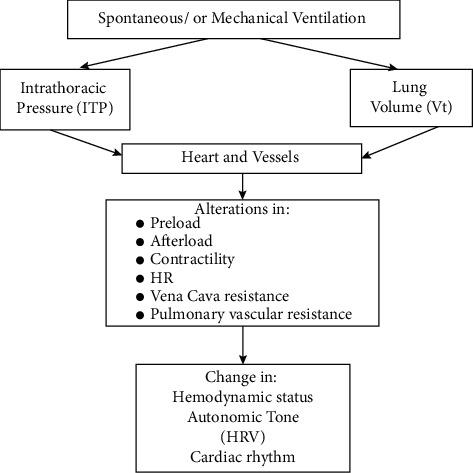
The cardiac and vascular function alterations with spontaneous and mechanical ventilation.

Both spontaneous and positive-pressure MV (which is currently the most commonly used MV in ICUs) induce alterations in ITP and lung volume above an end-expiratory baseline [20]. These alterations can independently influence the determinants of cardiovascular function: stroke volume (atrial filling or preload; the impedance to ventricular emptying or afterload, contractility) and heart rate (HR) [13]. Changes in ITP transfer to the intrathoracic structures, namely, the heart and pericardium, and the great vessels (veins and great arteries). The changes in the heart and great vessels lead to changes in the hemodynamic status, autonomic tone, and cardiac rhythm [13] (Figure 1).

Several hemodynamic effects of all forms of MV are similar despite variances in the mode of MV [21, 22]. ITP, however, decreases during spontaneous inspiration and increases during positive-pressure MV. Therefore, the primary reason for different hemodynamic responses seen during spontaneous and positive-pressure MV is related to the changes in ITP and the energy necessary to produce those changes [23–26]. At very high lung volumes, which are induced by MV, the expanding lungs compress the heart (particularly the cardiac fossa), limiting absolute cardiac volumes parallel to cardiac tamponade, except that with hyperinflation, both pericardial pressure and ITP increase by a similar amount [27].

### 1.2. Heart and Lung Interaction

The heart and lungs are closely coupled by their anatomical locations within the thorax and their responsibility to deliver the O_2_ requirements of cells and organs while excreting the CO_2_ byproduct of the metabolism [21]. In critically ill patients, if these two organ systems fail, either alone or in combination, the result is inadequate O_2_ delivery to the body along with expected tissue ischemia, progressive organ dysfunction, and if untreated, death. Therefore, reestablishment and maintenance of controlled cardiopulmonary function is an essential goal in the management of critically ill patients in ICUs [28]. Critically ill patients with a different diagnosis such as heart failure experience difficulty in gas exchange due to pulmonary edema and limited blood flow to the respiratory muscles [29]. Ventilation can alter cardiovascular function and stroke volume by increasing metabolic demands and autonomic tone by altering lung volume or VT, ITP [21, 28, 29].

### 1.3. Autonomic Tone

Autonomic tone neural processes probably play a primary role in all the long-term effects of ventilation on the cardiovascular system. Most of the immediate effects of MV on the cardiac function are secondary to changes in autonomic tone [30]. The lungs are richly enervated with somatic and autonomic fibers that originate, pass through, and end in the thorax. These networks mediate multiple homeostatic processes through the ANS altering immediate cardiovascular function [30, 31]. The most known of these networks are the vagally mediated HR changes during MV [31]. Inflation of the lung to normal VT (15 mL/kg) decelerates HR by a combination of both increased vagal tone and sympathetic withdrawal [32]. Sympathetic withdrawal also produces arterial vasodilation [21, 32]. This inflation-vasodilation response can reduce left ventricular (LV) contractility in both healthy volunteers and in ventricular-dependent patients with the initiation of high-frequency ventilation or hyperinflation [33]. This inflation-vasodilation response is assumed to be the cause of the initial hypotension seen when infants are placed on MV. It seems to be mediated partially by afferent vagal fibers because it is eliminated by selective vagal tone (vagotomy) [33]. These data suggest that lung inflation mediates its reflex on cardiovascular effects by controlling essential autonomic tone.

### 1.4. Cardiovascular Effects during Spontaneous Respiration

During spontaneous respiration, negative ITP induces a decrease in right atrial pressure (RAP) and an increase in right ventricular (RV) transmural pressure (i.e., the difference between cardiac intramural and extramural pressure) [21]. The decrease in RAP and the increase in RV transmural pressure subsequently increase the pressure gradient between the vena cava and RA for venous return to the heart [34]. The result of increased venous return to the right side of the heart causes an increase in IT vascular volume and RV diastolic filling volume [21]. The increase in IT vascular volume and RV diastolic filling volume increases RV end-diastolic volume, which results in an increase in stroke volume by the Frank–Starling mechanism (i.e., cardiac output increases or decreases in response to changes in HR or stroke volume) [34]. The Frank–Starling mechanism is one of the most important physiological principles for regulation of contractile performance. Changes in left ventricular (LV) preload tend to follow RV preload within 1-2 beats [21]. An increase in RV end-diastolic volume results in a decrease in LV diastolic compliance and end-diastolic volume through ventricular interdependence and leftward septal shift. LV afterload increases secondary to the increased LV transmural pressure [21].

As a result, LV stroke volume decrease, and a fall (<10 mmHg) in systolic blood pressure during inspiration is observed. The decrease in systolic blood pressure stimulates the baroreceptor reflex, which consequently increases sympathetic outflow to the heart, and a slight increase in HR occurs [35].

### 1.5. Cardiovascular Effects of Mechanical Ventilation

Unlike spontaneous respiration, positive-pressure MV results in an increase in ITP during inspiration and throughout the respiratory cycle if positive end-expiratory pressure (PEEP) or CPAP is used [25]. During inspiration, positive ITP is associated with decreases in stroke volume, including venous return and RV transmural pressure, causing a reduction preload [23, 35]. In addition, positive ITP and expanding lung volume compress the vena cava and RV, which in turn cause a decrease in RV preload [36]. At peak lung volume, especially in severe obstructive pulmonary disease with lung hyperinflation, or when PEEP is applied, pulmonary vascular resistance (PVR) is elevated and RV ejection is blocked [36, 37]. Given the tendency toward lower cardiac functions in some patients receiving MV and to increase blood pressure and maintain adequate cardiac output, the neurohormonal system responds to these hemodynamic alterations by secreting catecholamine, vasopressin, and rennin. Activation of the rennin-angiotensin system induces increases in angiotensin II and aldosterone levels [36].

The result of the increases of these neurohormones includes tachycardia, vasoconstriction, oliguria, retention of sodium and water, and subsequently increases in blood pressure. An increase in HR occurs as an initial response to lower cardiac outcomes. This can be seen in the beat-to-beat variations in HR that compensate for cyclical differences in RV and LV outputs [36, 37]. In brief, positive ITP that is associated with positive-pressure ventilation causes decreases in stroke volume, including in venous return, RV end-diastolic volume, and LV afterload. Decreases in preload and afterload cause a dramatic decrease in cardiac outcomes in patients with hypovolemia [36]. Decreases in preload and afterload may improve cardiac outcomes and ANS status (reflected by HRV, particularly as increased RSA or HF power).

### 1.6. Cardiovascular Effects of Weaning from Mechanical Ventilation

Transition from MV to spontaneous ventilation during the weaning process also produces hemodynamic alterations and ANS dysfunction (reflected by decreased HRV), especially in patients with underlying cardiovascular dysfunction [38]. ANS alterations can cause cardiac dysrhythmias depending on underlying cardiovascular function and adequacy of compensatory mechanisms [39]. On average, weaning accounts for 40% of the total duration of MV support [38, 40, 41]. During weaning from MV, different modes of spontaneous ventilation (including noninvasive methods) can be used that result in different hemodynamic responses dependent on ITP changes inherent to each [26, 42]. The hemodynamic responses to weaning include cardiovascular dysfunction, autonomic alteration, cardiac dysrhythmias, and myocardial ischemia [26].

During weaning, ITP decreases, and the degree of ITP reduction depends on the mode of MV used. The decrease in ITP may result in rapid increases in stoke volume (venous return, RV preload, and LV afterload) [26]. Additionally, during weaning, hypoventilation and alveolar hypoxia may occur and induce hypoxic pulmonary vasoconstriction, profoundly increasing RV afterload [43]. Alterations in preload and afterload that occur during weaning may induce acute cardiac mechanical changes and ANS alterations that may be arrhythmogenic [26]. Changes in ANS tone occur as a result of fluid shifts into the intrathoracic vascular compartment from changes in ITP [26]. The ANS compensates for these fluid shifts with an increase in sympathetic tone and a decrease in parasympathetic tone [13]. The result is a decrease in HRV and further increase in LV afterload. Sympathetic dominance is associated with negative effects on the cardiac function and leads to cardiac dysrhythmias [44]. Generally, patients with cardiovascular dysfunction and impaired compensatory mechanisms for alterations in stroke volume, HR, and ANS that occur during weaning may experience significant cardiac dysrhythmias [26].

### 1.7. Heart Rate Variability

HRV is the analysis of beat-to-beat variation in HR, which results, in large part, from ongoing changes in sympathetic and parasympathetic inputs to the sinoatrial node. It is related to the influences of the ANS during invasive and noninvasive MV [45]. The ANS is an important factor of the physiology of cardiac rhythm. A link between ANS alteration, particularly increased sympathetic activity and decreased parasympathetic activity (as reflected by decreased HRV), and the occurrence of dysrhythmias was demonstrated in numerous experimental and clinical studies [46].

### 1.8. Spectral Analysis of Heart Rate Variability

Frequency-based measure analysis, or spectral analysis of HRV, is a technique that provides discrete measures of the vagal as well as the sympathetic and parasympathetic modulation of the heart [47]. Frequency domain measures of HRV are defined according to the task force committee [48]. The HF (0.15–0.40 Hz) represents the vagal control to the heart [10], modulated by respiration [48, 49]. The LF (0.04–0.15 Hz) is more controversial and has contributions from both the vagal and sympathetic modulation of the heart [50]. However, many investigators proposed LF as an index of sympathetic modulation, and very low frequency power (0.03-0.04 Hz) represents the sympathetic activity. The ratio of LF to HF is used as an index of sympathovagal balance of the ANS [45, 47].

### 1.9. Heart Rate Variability during Spontaneous Respiration

There are some factors that possibly contribute to most of the measurement of frequency-based measures of HRV: the basal firing rate of nucleus ambiguous motor neurons in the brain (i.e., interbeat interval variation), respiratory patterns, and central respiratory drive [11]. Nucleus ambiguous motor neuron firing provides the strongest contribution, and correcting this for respiratory frequency does not appear to add additional explanatory power [51–53]. Respiratory parameters (e.g., depth and frequency) cause variations in HR [11, 54]; this is known as RSA. Changes in respiratory patterns can influence ITP, stroke volume, HR, and HRV [55–57]. In general, a decrease in respiratory frequency is associated with an increase in the heart rate [58]. RSA can be measured by capturing the HF power spectrum that corresponds with respiration as the parasympathetic nervous system operates using signaling mechanisms that can change HR in phase with respiration [59]. In light of this, the measurement of spontaneous respiration rate was previously recommended to ensure that vagal modulation does not occur outside the specified HF frequency band [60]. It is especially important to monitor this in populations known to have slower (e.g., athletes) or faster (e.g., children and premature infants) respiratory frequencies [61]. As breathing slower than a 0.15 Hz frequency substantially increases the observed power of RSA over that of typical breathing frequencies due to baroreflex recruitment, this confers a sizable impact on spectral HRV measures, highlighting the important link between respiration and HRV measurement [19]. Denver and colleagues [62], however, reported that respiration does not affect the HF component of HRV at rest in healthy participants.

**Figure 2:**
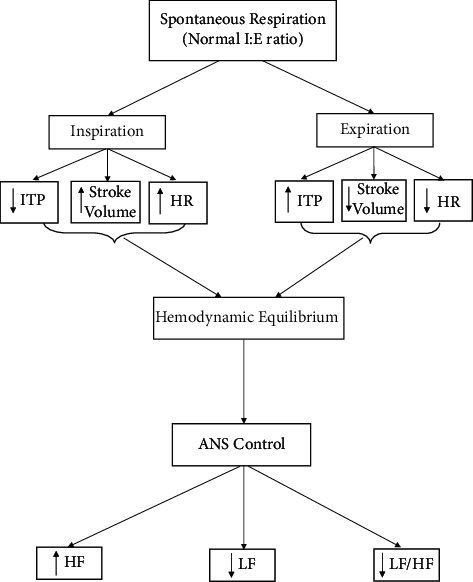
Heart rate variability during spontaneous respiration.

By reviewing the cardiovascular effect of spontaneous respiration, frequency-based measures of the HRV effect during spontaneous breathing can be evaluated. During spontaneous inspiration, there is a decrease in ITP and an increase in HR and stroke volume. While during spontaneous expiration, there is an increase in ITP and a decrease in HR and stroke volume. During normal *I*/*E* ratio (1 : 2 in rest or sleep state and 1 : 1 in exertion), there will be hemodynamic equilibrium and ANS control. When there is a normal respiratory pattern, the frequency-based measures of HRV are less affected and result in the higher parasympathetic nervous system (HF) and lower LF and LF/HF ratio [56, 57] (Figure 2).

### 1.10. Heart Rate Variability during Mechanical Ventilation

**Figure 3:**
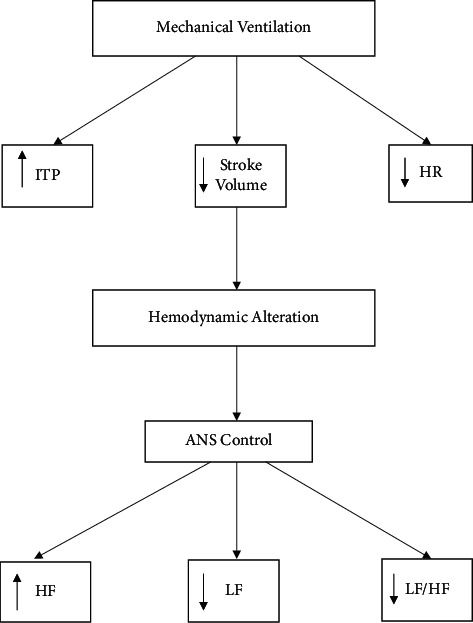
Heart rate variability during mechanical ventilation.

Porges suggested that abnormal ANS functioning characterized by decreased vagal modulation was in response to specific stresses such as MV. Thus, RSA quantification might be used for assessment of stress response [63]. RSA responses to different stressful stimuli showed large variability [64]. The RSA variations represent differences in the phasic pattern of vagal influences on HR [65]. Grossman and Taylor [65] referred to significant caveats: (1) breathing indices may affect the relation between RSA and parasympathetic HR regulation, (2) RSA recording is influenced by physical activity resulting in individual cardiac-vagal modulation, and (3) RSA amplitude is influenced by sympathetic modulation [65]. These issues should be considered in the correct interpretation of RSA as a marker of parasympathetic nervous system regulation of HR [64]. Based on literature review, Denver reported that RSA magnitude was not related to breathing parameters [62]. Several studies found that there is no significant effect of breathing parameters on RSA [66, 67]. Therefore, this area is still a matter of extensive discussion [67]. Based on the review about the cardiovascular effects during MV, it could be suggested that the frequency-based measure of HRV is characterized by increase in the parasympathetic nervous system and decrease in the sympathetic nervous system of the ANS in patients with hypervolemia. In contrast, HRV is characterized by a decrease in the parasympathetic nervous system and increase in the sympathetic nervous system in patients with hypovolemia. In summary, these data suggest that the influence of MV on HRV depends on the preexisting condition of cardiovascular status (Figure 3).

## 2. Heart Rate Variability and Weaning from Mechanical Ventilation

RSA is a normal phenomenon that refers to the cyclical variation in HR during the respiratory cycle, such that HR accelerates during inspiration and slows during expiration [68]. RSA is mainly mediated through changes in efferent vagal activity and is influenced by stretch receptors in the lung and by baroreceptors located in the carotid sinuses and aortic arch [65]. Lung inflation that occurs during inspiration stimulates the vagal nerves in the lung and induces reflex tachycardia. During expiration, increases in blood pressure stimulate baroreceptors; hence, reflex bradycardia is seen [69]. Weaning, the process of transition from MV to spontaneous ventilation, is associated with alterations in ANS tone, reflected by decreased HRV, especially in patients with underlying cardiovascular dysfunction and impaired compensatory mechanisms [70]. Alteration in HRV during weaning and its relation to weaning outcome was investigated in both nonhuman and human subjects [13–16].

**Figure 4:**
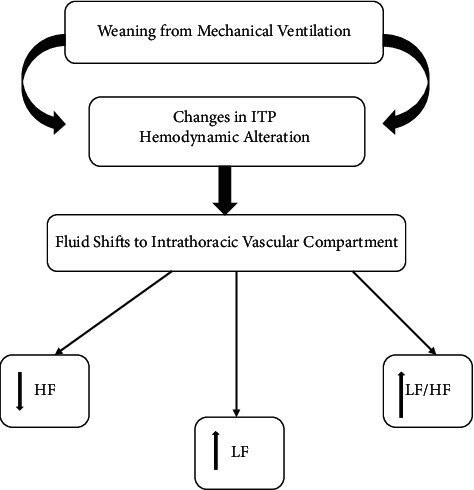
Heart rate variability and weaning from mechanical ventilation.

Huang et al. [71] analyzed HRV in 24 patients during transition from pressure support ventilation to spontaneous breathing trial [14–16, 71]. The investigators found a significant decrease in LF and HF of HRV in the group of patients who failed the weaning process, but not in the success group [14–16]. In an animal study, Frazier found a significant increase in very low frequency power and a significant decrease in HF power with exposure to a combination of pressure support and CPAP [13]. Changes in ANS tone during the weaning process occur because of fluid shifts into the intrathoracic vascular compartment from changes in ITP and hemodynamic alterations [13, 72]. The ANS compensates for these alternations with an increase in sympathetic tone (LF power) and a decrease in parasympathetic tone (HF power) or increase vagal withdrawal [13]. Thus, HRV is influenced by changing in respiratory patterns, ITP, VT, and hemodynamic alterations and causes impaired cardiac function and cardiac dysrhythmia [44]. The influence of MV on HRV is shown in Figures 3 and 4.

## 3. Conclusion

This review described the physiological influence of spontaneous and MV on HRV (particularly frequency-based measure). Despite the recognition that different types of MV are associated with alteration in ANS, there have been limited reports concerning the changes in the frequency-based measure of HRV (including HF, LF, and HF/LF ratio), which is a reliable method to detect ANS activity, particularly during the MV and weaning process [19]. MV is considered as an external stressor, which influences ANS status due to hemodynamic alterations in the lung-heart interaction (measured by frequency-based measure of HRV). The hemodynamic alterations (change in ITP, VT, and stroke volume alter autonomic tone) of ventilation are multiple, complex, and affect HRV (ANS tone). In patients' increased work of breathing or during respiratory distress, initiation of MV causes positive ITP, increasing stroke volume and decreasing HR. This will improve cardiac outcomes and oxygen delivery to the rest of the body by decreasing O_2_ consumption in case of respiratory distress. As a result, autonomic tone is reflected in HRV by increasing HF power (parasympathetic nervous system), decreasing LF (sympathetic nervous system), and decreasing LF/HF.

Transition from MV to spontaneous ventilation (the weaning process) produces hemodynamic alterations and ANS dysfunction (reflected by decreased HRV), especially in patients with underlying cardiovascular dysfunction. This will lead to a great change in HRV (RSA or HF) and may cause cardiac dysrhythmias. Understanding the physiological changes of frequency-based measure of heart rate variability, specific physiological changes of frequency-based measure of heart rate variability have been reviewed primarily during spontaneous respiration, mechanical ventilation, and weaning from mechanical ventilation based on the physiological analysis or review of the cardiovascular effect of spontaneous respiration, mechanical ventilation, and weaning process.

Of great scientific interest is the effect of spontaneous respiration, mechanical ventilation, and weaning from mechanical ventilation on frequency-based measure (or spectral analysis) of HRV by using data-based analysis for critically ill patients. In conclusion, ventilation is a ubiquitous phenomenon and its effects on HRV are a mandatory result. By understanding the effect of different types of ventilation (spontaneous, MV, and weaning process) on the cardiovascular system, the simple determinants of lung-heart interactions will be accurate, provide the most efficient treatment to patients in critical setting, and consider respiratory support as a confounding variable that might affect the high-frequency measure of HRV in respiratory distress patients with MV and during the weaning process.

## Abbreviations

ANS: Autonomic nervous system
AUC: Area under the curve
CPAP: Continuous positive airway pressure
HR: Heart rate
HRV: Heart rate variability
HF: High frequency
I\E: Inspiration/expiration ratio
ICU: Intensive care unit
ITP: Intrathoracic pressure
LF: Low frequency
LV: Left ventricular
MV: Mechanical ventilation
PEEP: Positive end-expiratory pressure
RAP: Right atrial pressure
RV: Right ventricular
TF: Total frequency
VT: Tidal volume.
